# Psychosocial and individual factors affecting Quality of Life (QoL) in patients suffering from Achilles tendinopathy: a systematic review

**DOI:** 10.1186/s12891-022-06090-2

**Published:** 2022-12-21

**Authors:** Josep Verges, Nina Martínez, Aina Pascual, Marco Bibas, Manel Santiña, Gil Rodas

**Affiliations:** 1 Osteoarthritis Foundation International OAFI, Barcelona, Spain; 2 Sociedad Española de Calidad Asistencial SECA, Oviedo, Spain; 3 Football Club Barcelona FCB, Barcelona, Spain

**Keywords:** Tendinopathy, Achilles tendon, Quality of life, Individual factors, Human factors, Psychosocial factors

## Abstract

**Background:**

Achilles tendinopathy (AT) is a joint condition that causes functional restrictions and pain. This condition negatively impacts patients' social connectedness and psychological well-being, reducing their quality of life (QoL). This review aims to summarise the current information on QoL in patients suffering from AT from different angles: compared to a healthy population, reported individual factors that influence it and the effects of some AT interventions on QoL.

**Methods:**

A systematic review was conducted at PubMed, Cochrane, Google Scholar, and PsycINFO using tendinopathy and QoL-related keywords up to November 2021. Articles were included if they compared QoL to demographic factors such as age or gender, lifestyle factors (physical activity levels), comorbidity factors (diabetes, obesity), and/or a control group.

**Results:**

Three hundred twenty-nine articles were reviewed; 23 met the inclusion criteria. SF-36, EQ-5D, and VISA-A were the most common instrument used. Patients with AT reported low QoL when compared to no AT population. When women were compared to men, women reported worse QoL. The patients who participated in different exercise programs (strengthening and stretching) showed improvements in QoL. Surgical AT intervention improved QoL, although results varied by age.

**Conclusion:**

AT has a substantial impact on QoL. In AT patients, QoL is also influenced by specific individual factors, including gender and physical activity. Exercise, education, and surgical treatment improve QoL. We suggest more research on AT patients to better understand the aspects leading to poor QoL.

**Supplementary Information:**

The online version contains supplementary material available at 10.1186/s12891-022-06090-2.

## Background

Tendinopathy is the most prevalent tendon disorder that causes pain and dysfunction. Achilles tendinopathy (AT) is a clinical condition characterised by pain and tendon thickening that affects the tendons and nearby structures, causing functional limitations and impaired load-bearing capacity [1, 2]. AT is one of the most frequent ankle and foot overuse injuries and a common cause of disability in activities that involve jumping, such as basketball and volleyball players, both recreational (ranging from 11.8% to 14.4%) and elite (32% to 45%) [3], AT is also found in people who participate in running activities [4] and professional dancers [5]. Still, it is not limited to athletic populations. It has an incidence of 83.3 (per 1000 athlete-year exposure) [6] and an incidence rate of 2.35/1000 subjects in the general population (21–60 years) [7].

AT has a poor prognosis, with a high incidence of chronicity and recurrence [8, 9]. The number of tendon-related procedures performed worldwide has been increasing over time, with approximately 30 million procedures every year. These translate into higher healthcare costs, estimated at around €140 billion [5, 10]. AT has been estimated to be €840 annually per patient [2]. These injuries are associated with high healthcare and socioeconomic costs, long-term postoperative rehabilitation, and loss of productivity [10].

The aetiology of AT is multifactorial, some studies indicates that the intrinsic factors could include biomechanical abnormalities of the lower extremity (leg length discrepancy hyperpronation, varus deformity of the forefoot, pes cavus and limited mobility of the subtalar joint), inflammatory arthropathies, corticosteroid use, diabetes, hypertension, obesity, gout, hyperostotic conditions, lipidaemias, aromatase inhibitors, quinolone antibiotics and age. These risk factors could be combined with extrinsic factors, including excessive mechanical overload, training errors (such as sudden increases or reductions in training levels, changes in type of loading, intensity of training sessions, duration of training) [11], and unsuitable sports equipment (such as innapropiate footwear) [4, 12, 13].

Pain caused by AT limits performing some activities and impaired load-bearing capacity associated with AT are assumed to decrease QoL [2]. However, the economic and social relevance of the problem needs to be adequately considered as the prevalence of AT is underestimated, specifically for active populations, such as athletes and workers with high physical activity, because AT is not easy to be diagnosed in an early stage [2, 14]. Moreover, not many studies with direct evidence support the effect on the patient’s QoL, the effectiveness of treatment and the economic implications of work disability [4, 15].

There is a need for advanced therapies to improve the social reintegration of patients with diseases of weight-bearing joints. There are various treatment options available for AT, but they are not very effective in treating symptoms, as two-thirds of the patients with new-onset AT remain symptomatic at 1- year follow- up [1]. Fast and good recovery is essential for these patients regarding social relationships. That is why assessing how AT affects patients' QoL (identity, social activities, pain and perceived fitness levels) is necessary to design effective disease management plans. Still, this information is not readily available for AT [2]. QoL is measured using self-reported questionnaires, referred to as Patient-Reported Outcome Measures (PROMs). PROMs reflect patients’ perceptions of their condition [16].

Therefore it becomes necessary to assess the impact of AT in the QoL, as this condition is highly prevalent, impacts patients' daily lives, and has a high economic impact due to work loss. This review aims to provide an international resource about the QoL of patients with AT and the associated factors such as demographic (age, sex, etc.) and individual factors (weight, physical activity, mental health, etc.), compared to a healthy population and some AT intervensions by analysing the available data in the literature.

## Methods

### Search strategy

Original articles were identified and listed using electronic searches of PubMed, Google Scholar, Cochrane, and PsycINFO databases. The literature review start date was unrestricted, and the end date was October 2022. languages not considered an exclusion criterion. The keywords used were "Achilles tendinopathy" AND "quality of life" OR "life quality" OR "well-being" OR "well-being" OR "short form 36" OR "Achilles injury " OR "AT" OR "AT-QoL" OR "FAOS" OR "assessment of the quality of life" OR "quality metrics" OR "quality of well-being" OR "SF-36" and all shorter forms and variations. The Preferred Reporting Items for Systematic Reviews and Meta-Analyses guidelines (PRISMA) and, although the protocol was not registered with PROSPERO, it is available on request [17].

### Inclusion and exclusion criteria

The tag flowchart used for inclusion and exclusion can be seen in Fig. S1. Abstracts or articles reporting original data on QoL of AT patients were included. Inclusion criteria were QoL associated with one or more demographic factors (e.g., age, gender), lifestyle factors (e.g., level of physical activity), or comorbidity factors (e.g., diabetes, obesity) and compared with a reference population or control group (without AT). There was no age, gender, language, or year of publication restriction. This review excluded review articles, protocols for clinical trials, preliminary results, commentaries, editorials, proceedings summaries, abstracts and instrument development summaries.

Articles that described unspecified AT or any study that combined AT patients with other types of patients and did not separately include, analyse, or report AT-specific data (e.g., a population defined as "Tendinopathy") were also excluded. Three reviewers independently assessed each reference against the prespecified inclusion and exclusion criteria. Then a two-stage process was performed: first, titles and abstracts were checked, and second, full-text articles. Any queries and inter-rater discrepancies were resolved during a consensus meeting.

### Data extraction

A single reviewer obtained the data for each eligible article using a pre-piloted extraction form. Study characteristics include publication details (author and year), participant characteristics (age, sex, body mass index [BMI], and the number of participants), instruments used, treatments applied in the intervention and control groups, and a summary of the main findings were extracted from the studies included. This data was then processed and reviewed by the three reviewers.

### Quality appraisal

A modified version of the Cochrane quality appraisal tool (National Institute of Health 2014) was used to assess the article's quality [18]. The assessment criteria are presented in Table 1. The strength of consistency between raters was not individually scored. One point was allocated for each item, with a maximum score of 13 (high-quality) and zero low quality). The studies were rated as low (0–4 points), moderate (5–9 points), or high (10–13 points).

**Table 1:** Quality Appraisal tool

Criteria	Yes	No	Other (CD, NR, NA)^a^
1. Was the research question or objective in this paper clearly stated?			
2. Was the study population specified and defined?			
3. Was there a justification for the instrument used?			
4. Was the design appropriate to meet the aims?			
5. Was the specifications of the subject group adequately given?			
6. Was a sample size justification, power description, or variance and effect estimates provided?			
7. Was the statistical methods adequately described?			
8. Was the data adequately described?			
9. Assessment of statistical significance			
10. Attention to potential biases			
11. Was the exposure assessed more than once over time?			
12. Comparison of results with previous reports			
13. Was there any implications in real life?			

^a^CD, cannot determine; *NA* not applicable, *NR* not reported

## Results

### Literature search results

**Fig. 1:**
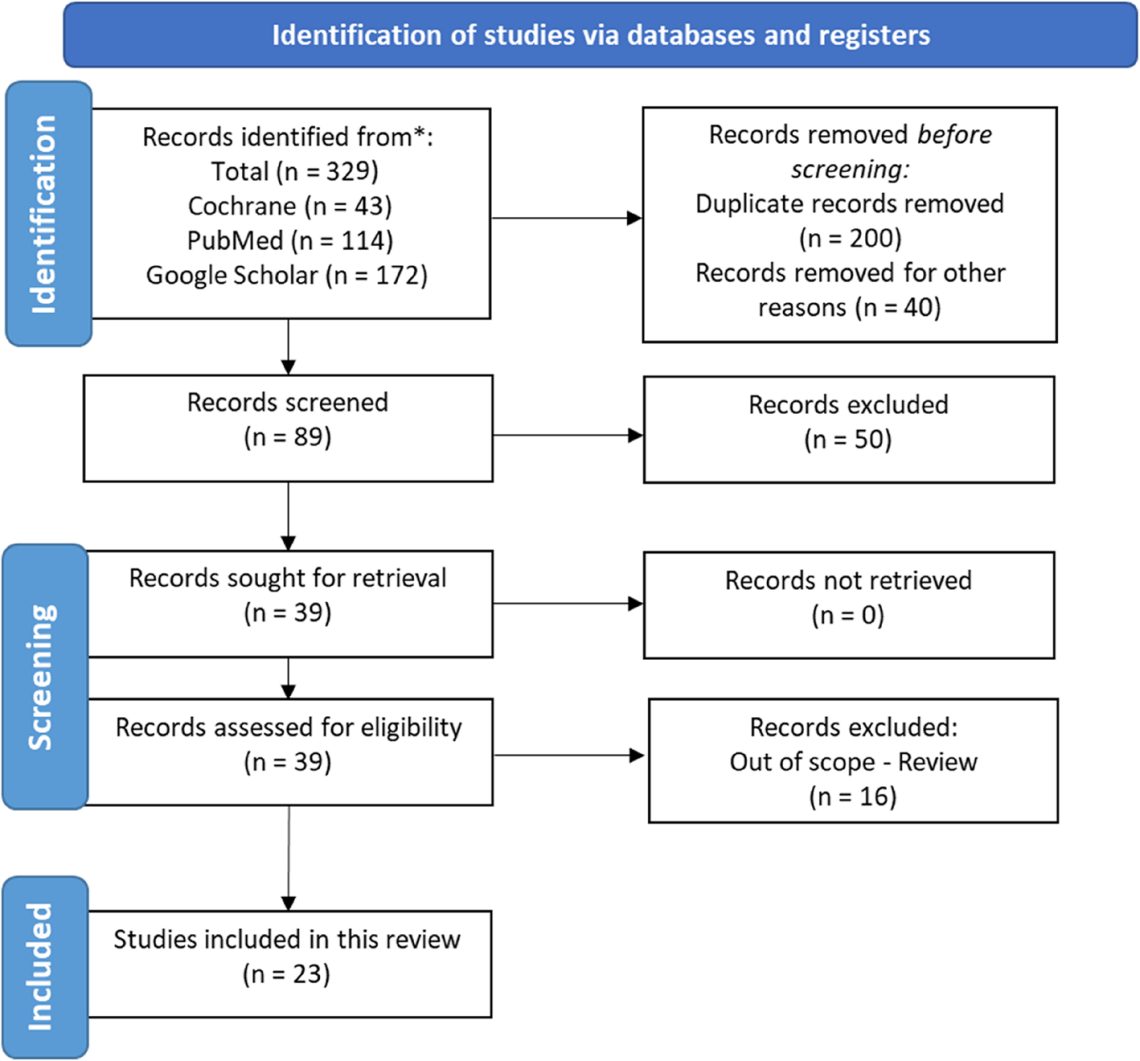
The flowchart of study selection

A total of 329 articles were initially identified (Fig. 1); 114 from PubMed, 43 from Cochrane, 172 from Google Scholar, and 0 from APA PsycINFO. A total of 89 articles were selected after the initial title, abstract screening, and removing duplicates. After manual searches and a full-text review, 39 articles were selected. The reference lists of all the included studies were examined to identify any additional studies that would meet the inclusion criteria. Twenty-three papers were included for final data extraction. All analyses were labelled by the first author and year of first publication. The year of publication ranged from 2004 to 2022.

### Characteristics of included studies

Study characteristic details are in Table 2. Most of the studies were conducted in the United States (*n* = 5) and the United Kingdom (*n* = 5), followed by Australia (*n* = 3), Netherlands (*n* = 2) and Sweden (*n* = 2), Germany (*n* = 2), Denmark (*n* = 1), Greece (*n* = 1), Ireland (*n* = 1), Norwich (*n* = 1). Thirty-eight percent (*n* = 8) of studies were cross-sectional in design, followed by other designs comprising 14.2% (*n* = 3) qualitative design, 14.2% (*n* = 3) retrospective, 9.5% (*n* = 2) prospective, 4.76% (*n* = 2) randomised controls trials, 4.76% (*n* = 1) population-based cohorts, 4.76% (*n* = 1) retrospective, 4.76% (*n* = 2) case–control, 4.76% (*n* = 2) pilot study. The SF-36 (*n* = 5) followed by FAOS (*n* = 4) were the main instruments used to assess QoL. All articles were rated from moderate to high-quality; no study scored lower than 7 points. Seventeen articles were classified as high quality, and six studies (23.8%) were moderate. The strength of consistency between raters was not scored. However, the average results are included in Table 2.

**Table 2:** Study characteristics

**References**	**Country**	**Study design**	**QoL instrument**	**Sample size with AT**	**Mean Age**	**Gender distribution**	**Symptoms’ duration**	**Quality assessment result**
Plinsinga et al. [19]	Australia	Case–control	EQ-5D, hospital anxiety and depression scale, TSK	75	AT: 45.7, Control: 41	AT: M: 17, F: 13, Control: M: 6, F:5		Moderate 9
Ceravolo et al. [20]	Australia	Exploratory study	AQoL-8D	92		M: 49, F:43		Moderate 8
Turner et al. [21]	Australia	Qualitative, interpretive description design	Semi-structured telephone interviews VISA-A	15	45.2	M:8, F:7	Eight months	Moderate 7
Nørregaard et al. [22]	Denmark		Modification KOOS	53	42		28.5 months	High 11
Petersen et al. [23]	Germany	Prospective Randomised Study	SF-36	100	42.5	M:60, F:40	7.4 months	High 11
Knobloch et al. [24]	Germany	Cohort Study	FAOS	86		M:38, F:25	7 months	High 10
Dedes et al. [25]	Greece	Cross-sectional	UoP-PFQ	130		M:60, F:70		High 11
Mc Auliffe et al. [26]	Ireland	Qualitative interpretive description design	Semi-structured telephone interviews, VISA-A	8	40	M:6, F: 2	20.5 months	Moderate 9
Opdam et al. [27]	Nether-lands	Retrospective	FAOS	59	50	M:18, F:27	45 months	High 11
Sleeswijk et al. [2]	Nether-lands	Cross-sectional	EQ-5D	80	50	M:39, F:41	15.7 months	High 11
Chester et al. [28]	Norwich	Pilot Study	VISA-A, EQ-5D	16	53.5	M: 11, F: 5	19 months	High 11
Roos et al. [29]	Sweden	Randomised study	FAOS	44	46	M:21, F:23	5.5 months	High 10
Alfredson et al. [30]	Sweden	Pilot study	SF-36	24	M:45, F:50	M:13, F:11	18 months	Moderate 8
Deans et al. [31]	UK	Prospective case series	FAOS	26	45.77	M:10, F:16		High 10
Maffulli et al. [32]	UK	Cross-sectional	VISA-A, EQ-5D	82	53.18	M:52, F:30		High 11
Kearney et al. [33]	UK	Multicenter randomised study	VISA-A, EQ-5D	240	52	M:102; F:138		High 10
Mallows et al. [34]	UK	Qualitative interpretive description design	Semi-structured interviews	10	49.2	M:6, F:4	12.9 months	High 11
Lewis et al. [35]	UK	Prospective comparative observational study	VISA-A, EQ-5D	320	52.1	M:125, F:195		High 11
Martin et al. [36]	US	Cross-sectional	SF-36	44	58.2	M: 18, F:26	24 months	Moderate 8
Chimenti et al. [37]	USA	Retrospective	SF-36	34	52.2		18 months	High 11
Corrigan et al. [38]	USA	Cross-sectional	VISA-A TSK	53	54.5	M:35, F:18	7 months	High 10
Phen et al. [39]	USA	Retrospective	SF-36	37	< 60: 49.1, > 60: 66.8	< 60: M:5, F:16, > 60: M:2, F:15		High 11
Chimeti et al. [40]	USA, Australia, Germany, India, New Zeland, UK	Cross-sectional	TSK-11	442	36.3	M:191, F:251		High 10

*AT* Achilles Tendinopathy, *M* Male, *F* Female, *EQ-5D* EuroQol 5 dimensions, *TSK* Tampa Scale for Kinesiophobia, *AQoL-8D* assessment of the quality of life-8-dimension, *VISA-A* Victorian Institute of Sport Assessment- Achilles questionnaire; KOOS: Knee Injury and Osteoarthritis Outcome Score; SF-36: Short Form 36, *FAOS* Foot and Ankle Outcome Score, *UoP-PFQ* University of Peloponnese Pain, Functionality and Quality of Life Questionnaire, *TSK-11* Tampa Scale for Kinesiophobia-11

### General characteristics of AT patients

The total population diagnosed with AT was 2070 people, of which 53.52% were women and 46.48% were men. All the articles included in this review included both men and women. The mean number of AT patients per study was 90, with a sample size varying between 8 [26] and 442 [37]. The mean age of patients was 49.1 and included ranges from 30 to 66.8. Six articles investigated midportion AT, five investigated insertional AT, four included patients with both midportion and insertional AT, and seven did not specify the location of AT. Concerning laterality, nine studies included both unilateral and bilateral AT, only one included unilateral AT, and fourteen did not specify.

### Difference in QoL between AT patients and healthy people

Table 3 shows the mean score from the different QoL instruments (PROMs) used in the articles included in this review. In the studies included in this review that compare QoL among AT and no AT patients, AT patients generally reported poor QoL compared with no AT population [19, 35, 36]. The results for the healthy population was 1.0 using the EQ-5D [19], 0.856 ± 0.175 using the EQ-5D-5L [35] and 50 (mean 10) for the Physical Component Summary (PCS) and 50 (mean10) for the Mental Component Summary (MCS) of the SF-36 [36]. In general, the PCS of SF-36 and SF-12 showed poorer results than the MCS. The mean VISA-A among the articles included was 56.70/100. A lower score indicates more symptoms and a more significant limitation of physical activity [24, 41].

**Table 3:** QoL outcomes by instrument

PROM	Mean	Median	IQR
AQoL-8D	78.7	78.7	78.7
EQ-5D	0.9	0.811	0.783
FAOS	42	37.5	33
MODIFIED KOOS	2.5	2.5	2.5
SF-12			
PCS	40.8	40.8	40.8
MCS	59.4	59.4	59.4
SF-16			
PCS	46.6	47.61	46.63
MCS	62.6	62.31	62
TSK	30	36	28.99
UoP-PFQ	2.65	2.65	2.65
VISA-A	56.7	57.70	52.35

*AQoL-8D* assessment of the quality of life-8-dimension, *EQ-5D* EuroQol 5 dimensions, *FAOS* Foot and AnkleOutcome Score, *IQR* interquartile range, *KOOS* Knee Injury and Osteoarthritis Outcome Score, *MCS* mental component summary, *PCS* physical component summary, *SF* Short Form, *TSK* Tampa Scale for Kinesiophobia, *UoP-PFQ* University of Peloponnese Pain, Functionality and Quality of Life Questionnaire, *VISA-A* Victorian Institute of Sport Assessment- Achilles questionnaire

### Associations between QoL and demographic factors in AT patients

The articles included in this review showed that some demographic factors impact QoL in patients with AT. Gender was found to affect QoL perception. Knobloch et al. [24] found that results were unfavourable among women compared to men. The results of FAOS QoL were 46 for women compared to 44 for men. Moreover, women did not benefit as much from eccentric or stretching training as men [22, 24]. Conversely, one study found no difference between genders [32]. Age was another factor that influenced patients’ perception of QoL. Phen et al. [39] reported that patient satisfaction was lower among patients older than 60 years of age compared to younger patients. Similarly, Knobloch et al. [24] found worse QoL and less improvement in women over 50 compared to the younger same-gender population.

Compared with the control population, QoL was reported to be worse, especially in the physical function, role-physical, bodily pain, and social functioning domains [19, 25, 30, 35, 36] and all EQ-5D domains except self-care. Lewis et al. [35] found that patients with AT aged < 55 had a significantly reduced QoL compared with the general UK population (EQ-5D mean 0.79 AT v. 0.906 no AT).

AT patients reported similar or worse QoL than individuals with other musculoskeletal diseases such as Rheumatoid Arthritis (RA), Osteoarthritis (OA), and fibromyalgia [2]. Moreover, lower QoL was associated with one or more comorbidities, specifically low back pain, high blood pressure, and diabetes [20]. Chimenti et al. [40] found that patients identified as Hispanic or Latino presented higher kinesiophobia levels compared to the group identified as Caucasian. Weight, height and BMI did not show a significant impact.

### Associations between QoL and psychosocial factors in AT patients

Mc Auliffe et al. [26] found that AT affected patients as they felt a loss of self-esteem. Moreover, patients reported frustration as practitioners could not explain AT and had different opinions. Turner et al. [21] said that 5 out of 15 patients felt frustration or dissatisfaction with their healthcare providers. Participants of this study reported that information is inconsistent and doctors don’t explain the condition in plain English. In the same study, all participants (*n* = 15) said that AT impacted their daily routine, and 11/15 reported frustration with their condition and how it limited their activities. AT patients also complain about their situation disrupting their daily activities, which affects their well-being and QoL [2, 32].

### Associations between QoL and physical activity in AT patients

It has been reported that AT is common in those who participate in sporting activities. However, it also affects less-active individuals. The studies included in this review show that QoL affects both patients with low and high activity levels, as both groups showed higher degrees of kinesiophobia. The loss of the ability to exercise was reported to have a significant impact on patients’ QoL [34]. However, those individuals who were active in sports before suffering from AT reported a stronger influence on their QoL after even non-interventional treatment [20, 29].

Some studies assessed the effectiveness of training programs such as eccentric and stretching exercises [22–24, 28, 29]. Stretching exercises showed some positive results in improving patients’ QoL after 52 weeks (< 0.05) [22]. A pilot study found no significant effects on QoL with eccentric exercise [28]. Eccentric exercises improved VISA-A score in males by 27%, from 63 ± 12 to 86 ± 13, and by 20% in females from 60 ± 14 to 75 ± 11 after 12 weeks [24]. The use of eccentric exercise and braces has been assessed, showing improvement in patients’ QoL (FAOS) from 33 ± 17 to 65 ± 27 for eccentric exercises, 32 ± 16 to 59 ± 21 for eccentric exercises and the use of a brace and 48 ± 15 to 62 ± 28 for the benefit of brace only [29]. Meanwhile, Petersen et al. [23] found that only function and pain categories of the SF-36 improved after 6, 12 and 54 weeks of eccentric training, AirHeel brace, and a combination of eccentric training and AirHeel brace.

### QoL and educational needs

Mallow et al. [34] conducted an interview and found that therapist understanding and empathy were essential for patients. Moreover, education was considered crucial, as well as good management of pain during exercise and a personalised approach with respect and understanding of each patient personal social circumstances and lifestyle. Patients also reported how AT has negatively impacted their self-identity and well-being and has caused body perception disturbance [23].

### Effects on interventions for AT patients

Conservative treatment for AT includes therapeutic ultrasound, shockwave intervention, platelet-rich plasma (PRP) injection and sham injection, and depending on the degeneration of the tendon, minimally invasive and open surgery could also be performed [4].

#### Shockwave and theraupeutic ultrasound

A study found that shockwave intervention had better results than the therapeutic ultrasound intervention showing a significant reduction in QoL impairment from 2.07 and reaching 0.00 after four weeks [25]. Similar results were found by Maffuli et al. [32], who found that QoL was enhanced after shockwave treatment. With significant effects on EQ-5D mobility, pain and usual activities (p,0.0001). On the other hand, PRP injection showed no significant differences in quality of life (EQ-5D-5L utility and VAS score) [33]. Deans et al. [31] showed that although the physical domain improved after Autologous-conditioned Plasma (ACP) injections combined with exercise and therapeutic ultrasonography, patients’ QoL did not show significant improvement as patients reported that they had not been able to resume the activities they were used to before the injury. Similarly, the use of ultrasound and colour doppler-guided surgery for insertional AT showed improvement in QoL, and the PCS improved from 42 (RP; SD 37)—64 (PF; SD 22) before surgery to 85 (RP; SD 28)—87 (RF; 14) at the one-year follow-up; the MCS increased from 62 (VT; SD 21)—82 (SF; SD 21) before surgery to 71 (VT; SD 23)—93 (SF; SD 18) at the one-year follow-up [30].

#### Surgical intervention

Some studies showed that surgical intervention and its impact on QoL depended on demographic variables and only improved physical components. Chimenti et al. [37] reported that only the physical component showed improvement after a percutaneous ultrasonic tenotomy( PCS 40.8 ± 9.4 44.0 ± 7.1), while no significant changes were found in the mental component (MCS 59.4 ± 5.2 59.8 ± 3.7). Patients with bilateral AT showed similar improvements after endoscopic treatment to unilateral patients (75 to 99 and 75 to 97 FAOS) except for activity in the daily living domain, while the median EQ-5D was 0.81 (IQR 0.71–1) and 1 (IQR 0.64–1), respectively [27]. Phen et al. [39] reported that after surgery, both PCS and MCS of SF-36 showed improvement from PCS 49.3 preoperative to 74.7 after 12 months and MCS 68.5 preoperative to 83.0 after 12 months post-surgery.

## Discussion

Articles about AT usually assess the effectiveness of exercise and conservative and invasive interventions; however, not many consider the impact AT has on patients' QoL. Some articles included QoL as isolated secondary measures that are not later taken into further consideration. The most recent studies may result from compliance with the minimum reporting standards for tendinopathy studies according to the international consensus (ICON) statement 9 core domains, including QoL [42]. As AT has been described to have a complex and often persistent nature, addressing other factors, in addition to treatment effectiveness related to pain and function scores, may be beneficial for a better comprehension of patients' needs and expectations. Thus, QoL should be considered an important aspect to assess and consider for AT patient management.

This systematic review aimed to summarise the available data reporting QoL measures in AT patients. Twenty-three articles were identified as reporting information on QoL associated with one other factor (demographic, lifestyle, or comorbidity), AT and control patients and/or intervention.

As shown in the articles included in this review, PROMs assessed how patients feel from their perspective. PROMs findings are of utmost importance for a more holistic treatment approach and to ensure health care services and procedures make a difference to a patient's health status and quality of life [16]. Most of the articles included in this review used the SF-36 followed by FAOS. However, creating and validating more PROMs specific to AT seems necessary, as the use of generic QoL instruments such as SF-36, SF-12, EQ-5D, and EQ-8D are not specific to AT, and this could affect the results by not being sensitive enough [42, 43].

Patients reported worse QoL in the physical aspects of SF-12 and SF-36, and low VISA-A scores were also found. Although VISA-A does not directly reflect QoL, it contains questions related to activities of daily living, functional mobility, gait, life participation and pain.

All studies reported that AT patients had worse QoL, with some factors having a more significant adverse effect on QoL than others. For example, demographic factors such as gender and age influenced QoL. These results are similar to the ones found for knee osteoarthritis (KOA) [44] and foot disorders [45]. Whereas weight, height and BMI did not show a significant impact. Women reported worse QoL than men. Similar findings have been reported for foot problems [45]. Moreover, comorbidities play an important role in the aggravation of AT symptoms and worse QoL.

The results of this review show that AT is associated with psychological distress and poor QoL. These results are similar to those found in other musculoskeletal disorders, such as KOA [44]. Evidence from other musculoskeletal disorders suggests the importance of understanding the potential implications of QoL factors on essential aspects such as prognosis and treatment adherence [46].

Social connectedness and how patients interact with their healthcare experts also impact QoL. AT patients are at risk of developing depression, kinestophobia, and pain catastrophising. This feeling of hopelessness can moderately or intensely reduce their ability to perform daily tasks. Some studies suggest that factors such as fear and self-efficacy are relevant for tendinopathy [19, 47].

Patients' social situations and context should be considered [24] when treating AT patients, as their improvement depends not only on their treatment as individuals but also as part of a socially connected environment. Community and patient organisations could help patients and improve communication between patients and healthcare providers. In fact, the more the patients engage and understand their condition, the better the result in terms of health outcomes and satisfaction [48]. Creating programs where patients participate in education and supervised exercise delivered by trained healthcare providers can improve pain intensity and QoL.

The need for good communication between patients and healthcare providers was also found to impact QoL [21, 26]. Wittink and Oosterhaven [49] highlight the importance of healthcare providers giving understandable information and avoiding medical jargon by embracing a more biopsychosocial approach. The use of this approach could contribute to reducing patients’ frustration and address their fears and beliefs [21, 26, 49].

Regarding physical activity, reducing physical activity impacts patients socially, affecting overall health and quality of life [24]. Results also showed that patients feared carrying out specific movements [38, 40]. Eccentric exercise has been reported as improving AT [50] due to neuromuscular changes. The articles included in this review also found that eccentric exercises improve patients’ QoL [23, 24]. On the other hand, surgery is a common treatment option when non-surgical options fail [51]. This review found that surgery has a positive impact on QoL.

To the best of our knowledge, this is the first systematic review focusing on QoL in AT patients. Studies were manually reviewed after the adoption of a broader search strategy. This study highlights the importance of understanding the priority of a patient's perspective about their condition. Also, we aimed to collect information on the perception of QoL in AT patients and understand how individual factors and social determinants of health can affect it. The search was broad, including all articles that reported QoL in AT regardless of whether QoL was a primary endpoint, as long as additional selection criteria were met.

## Limitations

There are limitations to acknowledge in this review. First, the description of sample size, data, etc., did not reflect the true methodological quality of the studies; thus, the high-quality scores should be interpreted with caution. Secondly, articles were included whether analysis was performed on QoL as the primary outcome; therefore, the sample size may have needed to have been sufficient to find differences when not all articles included QoL as the primary outcome. Thirdly, the methodological heterogeneity of the studies limited unbiased comparisons and quantitative syntheses, and the risk of bias was not calculated. More specific and standardised tools for assessing QoL in patients with AT are necessary. Finally, the present review was not registered on PROSPERO. Although registration is considered an advantage, it is not mandatory.

## Conclusion

AT is a problematic condition, both for the individual and healthcare providers. This review shows that AT patients have worse QoL than the general population and highlights the importance of recognising, assessing, and integrating QoL domains within the treatment paradigms and interventions to improve patient outcomes. It also shows the need for a new PROM for AT considered QoL. Moreover, this will give health professionals a better understanding of this condition which will positively impact AT patients’ management.

## Supplementary Information

**Additional file 1: Figure S1.** Flowchart used in the selection of the articles included in the study. The flowchart shows the sequence of criteria followed for the selection of the articles included. 

**Additional file 2: Table S1.** Search strings for the different databases.

## Data Availability

All data generated or analysed during this study are included in this published article.

## Abbreviations

ACT: Autologous-Conditioned Plasma
AQoL-8D: Assessment of the quality of life-8-dimension
AT: Achilles tendinopathy
BMI: Body Mass Index
EQ-5D: EuroQol 5 dimensions
F: Female
FAOS: Foot and Ankle Outcome Score
KOA: Knee Osteoarthritis
KOOS: Knee Injury and Osteoarthritis Outcome Score
ICON: International Consensus
IQR: Interquartile range
M: Male
MCS: Mental Component Summary
OA: Osteoarthritis
PCS: Physical Component Summary
PROM: Patient Reported Outcome
RA: Rheumatoid Arthritis
QoL: Quality of life
SF-36: Short Form
TSK: Tampa Scale for Kinesiophobia
UoP-PFQ: University of Peloponnese Pain, Functionality and Quality of Life Questionnaire
VISA-A: Victorian Institute of Sport Assessment- Achilles questionnaire
