# Supplementary material for: Psychosocial and individual factors affecting Quality of Life (QoL) in patients suffering from Achilles tendinopathy: a systematic review
Source: BMC Musculoskelet Disord. 2022 Dec 21;23:1114. doi: 10.1186/s12891-022-06090-2 (PMC9768977; doi:10.1186/s12891-022-06090-2)
Supplement: Supplementary file 1 — Additional file 1: Figure S1. Flowchart used in the selection of the articles included in the study. The flowchart shows the sequence of criteria followed for the selection of the articles included. [file 12891_2022_6090_MOESM1_ESM.docx]

**Additional file 1: Figure S1.** Flowchart used in the selection of the articles included in the study. The flowchart shows the sequence of criteria followed for the selection of the articles included.
