# Supplementary material for: Psychosocial and individual factors affecting Quality of Life (QoL) in patients suffering from Achilles tendinopathy: a systematic review
Source: BMC Musculoskelet Disord. 2022 Dec 21;23:1114. doi: 10.1186/s12891-022-06090-2 (PMC9768977; doi:10.1186/s12891-022-06090-2)
Supplement: Supplementary file 2 — Additional file 2: Table S1. Search strings for the different databases. [file 12891_2022_6090_MOESM2_ESM.docx]

| **Database** | **Search** |
| --- | --- |
| Cochrane | Achilles tendonitis" in Title Abstract Keyword AND quality life in Title Abstract Keyword OR "well-being" in Title Abstract Keyword OR "short form 36" in Title Abstract Keyword OR "SF-36" in Title Abstract Keyword OR "FAOS" in Title Abstract Keyword - (Word variations have been searched) |
| Google Scholar | intitle: ”Achilles tendinopathy” AND quality of life. |
| Pubmed | ((Achilles tendinopathy" AND "quality of life" OR "life quality" OR "well-being" OR "well-being" OR "short form 36" OR "Achilles injury " OR "AT" OR "AT-QoL" OR "FAOS" OR "assessment of the quality of life" OR "quality metrics" OR "quality of well-being" OR "SF-36 OR eq-5d OR eq-8d)) |

**Additional file 2: Table S1.** Search strings for the different databases
